# Supplementary material for: Comparison of lower-leg muscle activation and establishment of muscle activation patterns during single-leg stance under various instability conditions in healthy active subjects: a cross-sectional study
Source: PeerJ. 2025 May 23;13:e19461. doi: 10.7717/peerj.19461 (PMC12105616; doi:10.7717/peerj.19461)
Supplement: Supplemental Information 1 — Error bars: 95% CI. *Differences from the floor. † Differences from Rear. ‡ Differences from Inver. § Differences from Ever. # Differences from Sup. [file peerj-13-19461-s001.pdf]

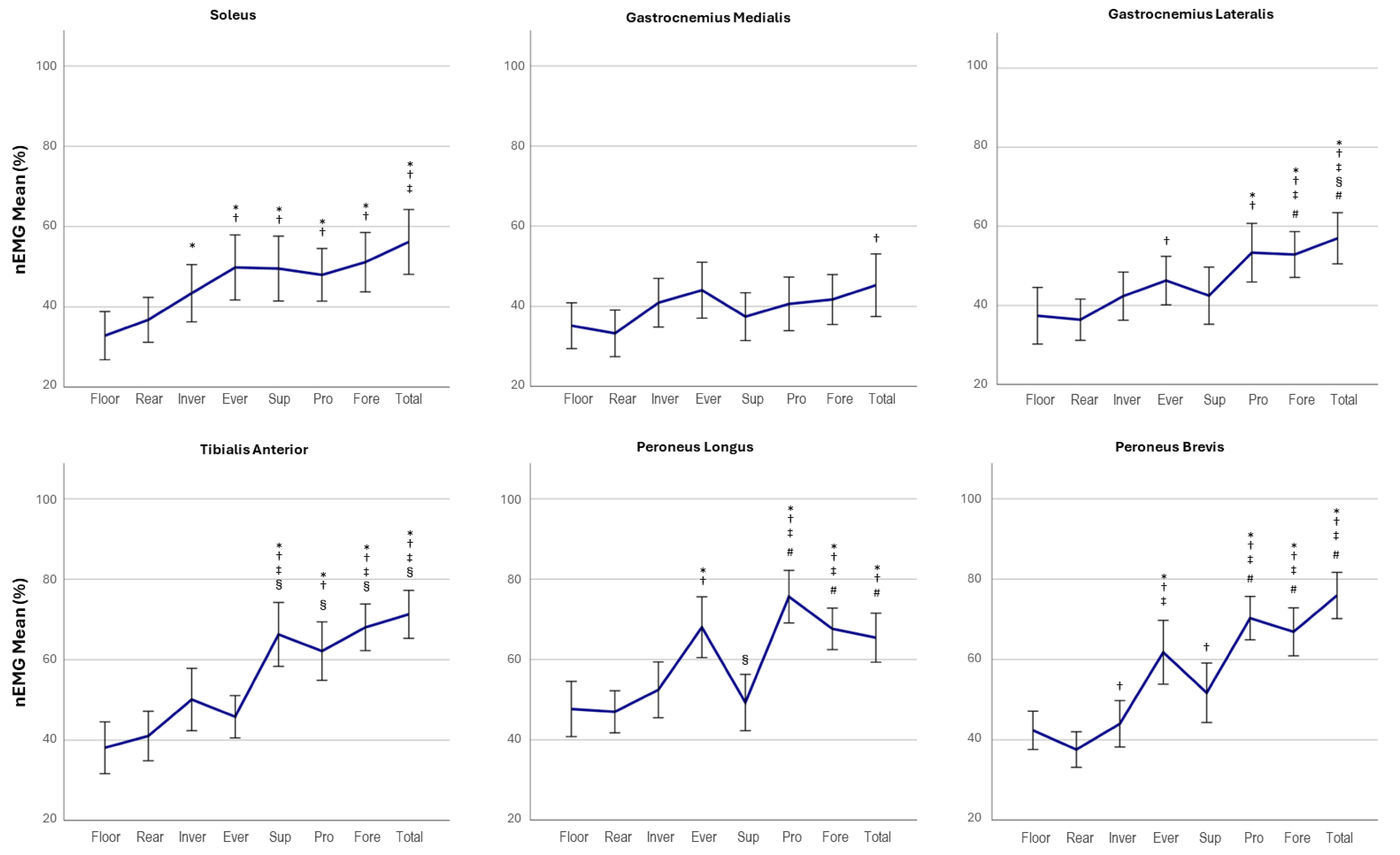

Soleus
Gastrocnemius Medialis
Gastrocnemius Lateralis
Tibialis Anterior
Peroneus Longus
Peroneus Brevis
nEMG Mean (%)
nEMG Mean (%)
100
80
60
40
20
Floor
Rear
Inver
Ever
Sup
Pro
Fore
Total
*
†
‡
§
#
