# Supplementary material for: Comparison of lower-leg muscle activation and establishment of muscle activation patterns during single-leg stance under various instability conditions in healthy active subjects: a cross-sectional study
Source: PeerJ. 2025 May 23;13:e19461. doi: 10.7717/peerj.19461 (PMC12105616; doi:10.7717/peerj.19461)
Supplement: Supplemental Information 2 [file peerj-13-19461-s002.docx]

Table 1 (supplementary). Between-condition nEMG differences (%) for all muscles. * Denotes statistically significant difference.

| **CONDITION** | floor | Rear | Inver | Ever | Sup | Pro | Fore | Total |
| --- | --- | --- | --- | --- | --- | --- | --- | --- |
| Total | 22.93* | 23.18* | 16.32* | 9.22 | 12.40* | 3.51 | 3.80 |  |
| Fore | 19.13* | 19.38* | 12.52* | 5.42 | 8.60* | -0.29 |  |  |
| Pro | 19.42* | 19.67* | 12.81* | 5.71 | 8.89* |  |  |  |
| Sup | 10.54* | 10.79* | 3.92 | -3.18 |  |  |  |  |
| Ever | 13.71* | 13.96* | 7.10 |  |  |  |  |  |
| Inver | 6.61 | 6.86* |  |  |  |  |  |  |
| Rear | -0.25 |  |  |  |  |  |  |  |
| floor |  |  |  |  |  |  |  |  |

Table 2 (supplementary). Between-muscle nEMG differences (%) for all conditions. * Denotes statistically significant difference.

| **MUSCLE** | SOL | GM | GL | TA | PL | PB |
| --- | --- | --- | --- | --- | --- | --- |
| PB | 10.36 | 16.53* | 10.31* | 0.97 | -2.82 |  |
| PL | 13.18 | 19.35* | 13.13* | 3.79 |  |  |
| TA | 9.39 | 15.56* | 9.35 |  |  |  |
| GL | 0.05 | 6.21 |  |  |  |  |
| GM | -6.17 |  |  |  |  |  |
| SOL |  |  |  |  |  |  |

Table 3 (supplementary). Mean differences for each muscle between conditions and its p-values. In bold those which are significant.

|  | | **Soleus** | | **Gastrocnemius medialis** | | **Gastrocnemius lateralis** | | **Tibialis Anterior** | | **Peroneus longus** | | **Peroneus brevis** | |
| --- | --- | --- | --- | --- | --- | --- | --- | --- | --- | --- | --- | --- | --- |
| **A** | **B** | **A-B** | ***p* value** | **A-B** | ***p* value** | **A-B** | ***p* value** | **A-B** | ***p* value** | **A-B** | ***p* value** | **A-B** | ***p* value** |
| **floor** | Rear | -3,951 | 0,754 | 1,899 | 1,000 | 0,992 | 1,000 | -2,929 | 1,000 | 0,698 | 1,000 | 4,798 | 1,000 |
|  | Inver | -10,581^*^ | **0,004** | -5,732 | 0,656 | -4,963 | 1,000 | -11,992 | 0,351 | -4,777 | 1,000 | -1,616 | 1,000 |
|  | Ever | -16,999^*^ | **<0.001** | -8,863 | 0,562 | -8,902 | 0,222 | -7,714 | 0,539 | -20,358^*^ | **<0.001** | -19,431^*^ | **<0.001** |
|  | Sup | -16,724^*^ | **<0.001** | -2,269 | 1,000 | -5,081 | 1,000 | -28,185^*^ | **<0.001** | -1,609 | 1,000 | -9,341 | 0,931 |
|  | Pro | -15,156^*^ | **<0.001** | -5,445 | 1,000 | -15,955^*^ | **0,005** | -24,040^*^ | **<0.001** | -27,981^*^ | **<0.001** | -27,934^*^ | **<0.001** |
|  | Fore | -18,319^*^ | **<0.001** | -6,545 | 0,808 | -15,485^*^ | **0,001** | -29,955^*^ | **<0.001** | -19,960^*^ | **0,001** | -24,524^*^ | **<0.001** |
|  | Total | -23,358^*^ | **<0.001** | -10,110 | 0,499 | -19,593^*^ | **<0.001** | -33,190^*^ | **<0.001** | -17,759^*^ | **0,004** | -33,581^*^ | **<0.001** |
|  | | | | | | | | | | | | | |
| **Rear** | floor | 3,951 | 0,754 | -1,899 | 1,000 | -0,992 | 1,000 | 2,929 | 1,000 | -0,698 | 1,000 | -4,798 | 1,000 |
|  | Inver | -6,630 | 0,064 | -7,631 | 0,155 | -5,955 | 0,687 | -9,063 | 0,274 | -5,475 | 1,000 | -6,414^*^ | **0,032** |
|  | Ever | -13,049^*^ | **0,004** | -10,762 | 0,099 | -9,893^*^ | **0,028** | -4,785 | 1,000 | -21,056^*^ | **<0.001** | -24,229^*^ | **<0.001** |
|  | Sup | -12,773^*^ | **<0.001** | -4,168 | 1,000 | -6,072 | 1,000 | -25,256^*^ | **<0.001** | -2,307 | 1,000 | -14,139^*^ | **0,004** |
|  | Pro | -11,205^*^ | **0,002** | -7,344 | 0,099 | -16,947^*^ | **<0.001** | -21,111^*^ | **<0.001** | -28,679^*^ | **<0.001** | -32,731^*^ | **<0.001** |
|  | Fore | -14,369^*^ | **<0.001** | -8,444 | 0,060 | -16,477^*^ | **<0.001** | -27,026^*^ | **<0.001** | -20,658^*^ | **<0.001** | -29,321^*^ | **<0.001** |
|  | Total | -19,407^*^ | **<0.001** | -12,009^*^ | **0,041** | -20,584^*^ | **<0.001** | -30,261^*^ | **<0.001** | -18,457^*^ | **0,002** | -38,378^*^ | **<0.001** |
|  | | | | | | | | | | | | | |
| **Inver** | floor | 10,581^*^ | **0,004** | 5,732 | 0,656 | 4,963 | 1,000 | 11,992 | 0,351 | 4,777 | 1,000 | 1,616 | 1,000 |
|  | Rear | 6,630 | 0,064 | 7,631 | 0,155 | 5,955 | 0,687 | 9,063 | 0,274 | 5,475 | 1,000 | 6,414^*^ | **0,032** |
|  | Ever | -6,418 | 1,000 | -3,131 | 1,000 | -3,939 | 1,000 | 4,278 | 1,000 | -15,581 | 0,121 | -17,815^*^ | **0,002** |
|  | Sup | -6,143 | 0,656 | 3,464 | 1,000 | -0,118 | 1,000 | -16,192^*^ | **0,003** | 3,168 | 1,000 | -7,725 | 0,334 |
|  | Pro | -4,575 | 1,000 | 0,288 | 1,000 | -10,992 | 0,070 | -12,048 | 0,237 | -23,205^*^ | **0,002** | -26,317^*^ | **<0.001** |
|  | Fore | -7,739 | 0,192 | -0,812 | 1,000 | -10,522^*^ | **0,016** | -17,963^*^ | **0,005** | -15,183^*^ | **0,010** | -22,908^*^ | **<0.001** |
|  | Total | -12,777^*^ | **0,011** | -4,378 | 1,000 | -14,630^*^ | **0,001** | -21,198^*^ | **0,002** | -12,982 | 0,065 | -31,965^*^ | **<0.001** |
|  | | | | | | | | | | | | | |
| **Ever** | floor | 16,999^*^ | **<0.001** | 8,863 | 0,562 | 8,902 | 0,222 | 7,714 | 0,539 | 20,358^*^ | **<0.001** | 19,431^*^ | **<0.001** |
|  | Rear | 13,049^*^ | **0,004** | 10,762 | 0,099 | 9,893^*^ | **0,028** | 4,785 | 1,000 | 21,056^*^ | **<0.001** | 24,229^*^ | **<0.001** |
|  | Inver | 6,418 | 1,000 | 3,131 | 1,000 | 3,939 | 1,000 | -4,278 | 1,000 | 15,581 | 0,121 | 17,815^*^ | **0,002** |
|  | Sup | 0,275 | 1,000 | 6,595 | 1,000 | 3,821 | 1,000 | -20,471^*^ | **0,001** | 18,749^*^ | **0,032** | 10,090 | 1,000 |
|  | Pro | 1,843 | 1,000 | 3,419 | 1,000 | -7,054 | 0,820 | -16,326^*^ | **0,002** | -7,623 | 0,307 | -8,502 | 0,517 |
|  | Fore | -1,320 | 1,000 | 2,319 | 1,000 | -6,583 | 0,564 | -22,241^*^ | **<0.001** | 0,398 | 1,000 | -5,092 | 1,000 |
|  | Total | -6,359 | 1,000 | -1,247 | 1,000 | -10,691^*^ | **0,023** | -25,476^*^ | **<0.001** | 2,599 | 1,000 | -14,150 | 0,236 |
|  | | | | | | | | | | | | | |
| **Sup** | floor | 16,724^*^ | **<0.001** | 2,269 | 1,000 | 5,081 | 1,000 | 28,185^*^ | **<0.001** | 1,609 | 1,000 | 9,341 | 0,931 |
|  | Rear | 12,773^*^ | **<0.001** | 4,168 | 1,000 | 6,072 | 1,000 | 25,256^*^ | **<0.001** | 2,307 | 1,000 | 14,139^*^ | **0,004** |
|  | Inver | 6,143 | 0,656 | -3,464 | 1,000 | 0,118 | 1,000 | 16,192^*^ | **0,003** | -3,168 | 1,000 | 7,725 | 0,334 |
|  | Ever | -0,275 | 1,000 | -6,595 | 1,000 | -3,821 | 1,000 | 20,471^*^ | **0,001** | -18,749^*^ | **0,032** | -10,090 | 1,000 |
|  | Pro | 1,568 | 1,000 | -3,176 | 1,000 | -10,875 | 0,072 | 4,145 | 1,000 | -26,372^*^ | **<0.001** | -18,593^*^ | **0,008** |
|  | Fore | -1,596 | 1,000 | -4,276 | 0,400 | -10,404^*^ | **0,004** | -1,771 | 1,000 | -18,351^*^ | **<0.001** | -15,183^*^ | **0,001** |
|  | Total | -6,634 | 0,269 | -7,842 | 0,696 | -14,512^*^ | **0,003** | -5,005 | 1,000 | -16,150^*^ | **0,006** | -24,240^*^ | **<0.001** |
|  | | | | | | | | | | | | | |
| **Pro** | floor | 15,156^*^ | **<0.001** | 5,445 | 1,000 | 15,955^*^ | **0,005** | 24,040^*^ | **<0.001** | 27,981^*^ | **<0.001** | 27,934^*^ | **<0.001** |
|  | Rear | 11,205^*^ | **0,002** | 7,344 | 0,099 | 16,947^*^ | **<0.001** | 21,111^*^ | **<0.001** | 28,679^*^ | **<0.001** | 32,731^*^ | **<0.001** |
|  | Inver | 4,575 | 1,000 | -0,288 | 1,000 | 10,992 | 0,070 | 12,048 | 0,237 | 23,205^*^ | **0,002** | 26,317^*^ | **<0.001** |
|  | Ever | -1,843 | 1,000 | -3,419 | 1,000 | 7,054 | 0,820 | 16,326^*^ | **0,002** | 7,623 | 0,307 | 8,502 | 0,517 |
|  | Sup | -1,568 | 1,000 | 3,176 | 1,000 | 10,875 | 0,072 | -4,145 | 1,000 | 26,372^*^ | **<0.001** | 18,593^*^ | **0,008** |
|  | Fore | -3,164 | 1,000 | -1,100 | 1,000 | 0,470 | 1,000 | -5,915 | 1,000 | 8,022 | 0,606 | 3,410 | 1,000 |
|  | Total | -8,202 | 0,288 | -4,666 | 1,000 | -3,637 | 1,000 | -9,150 | 0,311 | 10,222 | 0,546 | -5,647 | 1,000 |
|  | | | | | | | | | | | | | |
| **Fore** | floor | 18,319^*^ | **<0.001** | 6,545 | 0,808 | 15,485^*^ | **0,001** | 29,955^*^ | **<0.001** | 19,960^*^ | **0,001** | 24,524^*^ | **<0.001** |
|  | Rear | 14,369^*^ | **<0.001** | 8,444 | 0,060 | 16,477^*^ | **<0.001** | 27,026^*^ | **<0.001** | 20,658^*^ | **<0.001** | 29,321^*^ | **<0.001** |
|  | Inver | 7,739 | 0,192 | 0,812 | 1,000 | 10,522^*^ | **0,016** | 17,963^*^ | **0,005** | 15,183^*^ | **0,010** | 22,908^*^ | **<0.001** |
|  | Ever | 1,320 | 1,000 | -2,319 | 1,000 | 6,583 | 0,564 | 22,241^*^ | **<0.001** | -0,398 | 1,000 | 5,092 | 1,000 |
|  | Sup | 1,596 | 1,000 | 4,276 | 0,400 | 10,404^*^ | **0,004** | 1,771 | 1,000 | 18,351^*^ | **0,000** | 15,183^*^ | **0,001** |
|  | Pro | 3,164 | 1,000 | 1,100 | 1,000 | -0,470 | 1,000 | 5,915 | 1,000 | -8,022 | 0,606 | -3,410 | 1,000 |
|  | Total | -5,038 | 1,000 | -3,565 | 1,000 | -4,108 | 1,000 | -3,234 | 1,000 | 2,201 | 1,000 | -9,057 | 0,353 |
|  | | | | | | | | | | | | | |
| **Total** | floor | 23,358^*^ | **<0.001** | 10,110 | 0,499 | 19,593^*^ | **<0.001** | 33,190^*^ | **<0.001** | 17,759^*^ | **0,004** | 33,581^*^ | **<0.001** |
|  | Rear | 19,407^*^ | **<0.001** | 12,009^*^ | **0,041** | 20,584^*^ | **<0.001** | 30,261^*^ | **<0.001** | 18,457^*^ | **0,002** | 38,378^*^ | **<0.001** |
|  | Inver | 12,777^*^ | **0,011** | 4,378 | 1,000 | 14,630^*^ | **0,001** | 21,198^*^ | **0,002** | 12,982 | 0,065 | 31,965^*^ | **<0.001** |
|  | Ever | 6,359 | 1,000 | 1,247 | 1,000 | 10,691^*^ | **0,023** | 25,476^*^ | **<0.001** | -2,599 | 1,000 | 14,150 | 0,236 |
|  | Sup | 6,634 | 0,269 | 7,842 | 0,696 | 14,512^*^ | **0,003** | 5,005 | 1,000 | 16,150^*^ | **0,006** | 24,240^*^ | **<0.001** |
|  | Pro | 8,202 | 0,288 | 4,666 | 1,000 | 3,637 | 1,000 | 9,150 | 0,311 | -10,222 | 0,546 | 5,647 | 1,000 |
|  | Fore | 5,038 | 1,000 | 3,565 | 1,000 | 4,108 | 1,000 | 3,234 | 1,000 | -2,201 | 1,000 | 9,057 | 0,353 |

Table 4 (supplementary). Significant differences between conditions for each muscle with effect sizes (Cohen’s *d*).

| **nEMG** | **Comparisons** | **Difference (%)** | **p-value** | **Effect Size (d)** |
| --- | --- | --- | --- | --- |
| **SOL nEMG** | Total vs Floor | 23.36% | p<0.001 | d=1.37 |
|  | Total vs Rear | 19.41% | p<0.001 | d=1.35 |
|  | Total vs Inver | 12.78% | p=0.011 | d=0.73 |
|  | Fore vs Floor | 18.32% | p<0.001 | d=1.56 |
|  | Fore vs Rear | 14.37% | p<0.001 | d=1.34 |
|  | Pro vs Floor | 15.16% | p<0.001 | d=1.08 |
|  | Pro vs Rear | 11.21% | p=0.002 | d=0.86 |
|  | Sup vs Floor | 16.72% | p<0.001 | d=1.04 |
|  | Sup vs Rear | 12.77% | p=0.002 | d=1.01 |
|  | Ever vs Floor | 17.00% | p<0.001 | d=1.07 |
|  | Ever vs Rear | 13.05% | p=0.004 | d=0.79 |
|  | Inver vs Floor | 10.58% | p=0.004 | d=0.80 |
| **GM nEMG** | Total vs Rear | 12.01% | p=0.041 | d=0.64 |
| **GL nEMG** | Total vs Floor | 19.59% | p<0.001 | d=1.18 |
|  | Total vs Rear | 20.58% | p<0.001 | d=1.43 |
|  | Total vs Inver | 14.63% | p<0.001 | d=0.92 |
|  | Total vs Ever | 10.69% | p=0.023 | d=0.68 |
|  | Total vs Sup | 14.51% | p=0.003 | d=0.82 |
|  | Fore vs Floor | 15.49% | p<0.001 | d=0.88 |
|  | Fore vs Rear | 16.48% | p<0.001 | d=1.22 |
|  | Fore vs Inver | 10.52% | p=0.016 | d=0.71 |
|  | Fore vs Sup | 10.40% | p=0.004 | d=0.80 |
|  | Pro vs Floor | 15.96% | p=0.005 | d=0.78 |
|  | Pro vs Rear | 16.95% | p<0.001 | d=1.02 |
|  | Ever vs Rear | 9.89% | p=0.028 | d=0.67 |
| **TA nEMG** | Total vs Floor | 33.19% | p<0.001 | d=1.86 |
|  | Total vs Rear | 30.26% | p<0.001 | d=1.60 |
|  | Total vs Inver | 21.20% | p=0.002 | d=0.84 |
|  | Total vs Ever | 25.48% | p<0.001 | d=1.80 |
|  | Fore vs Floor | 29.96% | p<0.001 | d=1.65 |
|  | Fore vs Rear | 27.03% | p<0.001 | d=1.36 |
|  | Fore vs Inver | 17.96% | p=0.005 | d=0.78 |
|  | Fore vs Ever | 22.24% | p<0.001 | d=1.29 |
|  | Pro vs Floor | 24.04% | p<0.001 | d=1.10 |
|  | Pro vs Rear | 21.11% | p<0.001 | d=1.16 |
|  | Pro vs Ever | 16.33% | p=0.002 | d=0.86 |
|  | Sup vs Floor | 28.19% | p<0.001 | d=1.11 |
|  | Sup vs Rear | 25.26% | p<0.001 | d=1.26 |
|  | Sup vs Inver | 16.19% | p=0.003 | d=0.82 |
|  | Sup vs Ever | 20.47% | p<0.001 | d=0.92 |
| **PL nEMG** | Total vs Floor | 17.76% | p=0.004 | d=0.80 |
|  | Total vs Rear | 18.46% | p=0.002 | d=0.87 |
|  | Total vs Sup | 16.15% | p=0.006 | d=0.78 |
|  | Fore vs Floor | 19.96% | p=0.001 | d=0.90 |
|  | Fore vs Rear | 20.66% | p<0.001 | d=1.28 |
|  | Fore vs Inver | 15.18% | p=0.010 | d=0.74 |
|  | Fore vs Sup | 18.35% | p<0.001 | d=1.02 |
|  | Pro vs Floor | 27.98% | p<0.001 | d=1.27 |
|  | Pro vs Rear | 28.68% | p<0.001 | d=1.47 |
|  | Pro vs Inver | 23.21% | p=0.002 | d=0.86 |
|  | Pro vs Sup | 26.37% | p<0.001 | d=1.00 |
|  | Ever vs Floor | 20.36% | p<0.001 | d=1.08 |
|  | Ever vs Rear | 21.06% | p<0.001 | d=1.00 |
|  | Ever vs Sup | 18.75% | p=0.032 | d=0.66 |
| **PB nEMG** | Total vs Floor | 33.58% | p<0.001 | d=1.68 |
|  | Total vs Rear | 38.38% | p<0.001 | d=2.21 |
|  | Total vs Inver | 31.97% | p<0.001 | d=1.80 |
|  | Total vs Sup | 24.24% | p<0.001 | d=1.13 |
|  | Fore vs Floor | 24.52% | p<0.001 | d=1.20 |
|  | Fore vs Rear | 29.32% | p<0.001 | d=2.15 |
|  | Fore vs Inver | 22.91% | p<0.001 | d=1.78 |
|  | Fore vs Sup | 15.18% | p=0.001 | d=0.93 |
|  | Pro vs Floor | 27.93% | p<0.001 | d=1.52 |
|  | Pro vs Rear | 32.73% | p<0.001 | d=2.08 |
|  | Pro vs Inver | 26.32% | p<0.001 | d=1.45 |
|  | Pro vs Sup | 18.59% | p=0.008 | d=0.75 |
|  | Sup vs Rear | 14.14% | p=0.004 | d=0.80 |
|  | Ever vs Floor | 19.43% | p<0.001 | d=1.17 |
|  | Ever vs Rear | 24.23% | p<0.001 | d=1.15 |
|  | Ever vs Inver | 17.82% | p=0.002 | d=0.84 |
|  | Inver vs Rear | 6.64% | p=0.032 | d=0.66 |

Table 5 (supplementary). Mean differences for each condition between muscles and its p-values. In bold those which are significant.

|  | | **floor** | | **Rear** | | **Inver** | | **Ever** | | **Sup** | | **Pro** | | **Fore** | | **Total** | |
| --- | --- | --- | --- | --- | --- | --- | --- | --- | --- | --- | --- | --- | --- | --- | --- | --- | --- |
| **A** | **B** | **A-B** | ***p* value** | **A-B** | ***p* value** | **A-B** | ***p* value** | **A-B** | ***p* value** | **A-B** | ***p* value** | **A-B** | ***p* value** | **A-B** | ***p* value** | **A-B** | ***p* value** |
| **SOL** | GM | -2,336 | 1,000 | 3,514 | 1,000 | 2,513 | 1,000 | 5,800 | 1,000 | 12,119 | 0,298 | 7,376 | 1,000 | 9,439 | 0,401 | 10,912 | 0,723 |
|  | GL | -4,559 | 1,000 | 0,384 | 1,000 | 1,059 | 1,000 | 3,539 | 1,000 | 7,084 | 1,000 | -5,358 | 1,000 | -1,724 | 1,000 | -0,794 | 1,000 |
|  | TA | -5,277 | 1,000 | -4,256 | 1,000 | -6,689 | 1,000 | 4,008 | 1,000 | -16,738 | 0,175 | -14,161 | 0,113 | -16,913^*^ | **0,014** | -15,109 | 0,078 |
|  | PL | -14,847^*^ | **0,014** | -10,199 | 0,302 | -9,043 | 1,000 | -18,206^*^ | **0,024** | 0,267 | 1,000 | -27,673^*^ | **0,000** | -16,488 | 0,062 | -9,249 | 1,000 |
|  | PB | -9,542 | 0,321 | -0,793 | 1,000 | -0,577 | 1,000 | -11,974 | 0,406 | -2,159 | 1,000 | -22,319^*^ | **0,000** | -15,746^*^ | **0,045** | -19,765^*^ | **0,005** |
|  | | | | | | | | | | | | | | | | | |
| **GM** | SOL | 2,336 | 1,000 | -3,514 | 1,000 | -2,513 | 1,000 | -5,800 | 1,000 | -12,119 | 0,298 | -7,376 | 1,000 | -9,439 | 0,401 | -10,912 | 0,723 |
|  | GL | -2,223 | 1,000 | -3,130 | 1,000 | -1,453 | 1,000 | -2,261 | 1,000 | -5,035 | 1,000 | -12,734 | 0,063 | -11,163^*^ | **0,049** | -11,705 | 0,096 |
|  | TA | -2,941 | 1,000 | -7,769 | 0,918 | -9,201 | 0,266 | -1,792 | 1,000 | -28,858^*^ | **0,000** | -21,537^*^ | **0,000** | -26,352^*^ | **0,000** | -26,021^*^ | **0,000** |
|  | PL | -12,512 | 0,109 | -13,713^*^ | **0,015** | -11,556 | 0,314 | -24,006^*^ | **0,000** | -11,852 | 0,152 | -35,048^*^ | **0,000** | -25,927^*^ | **0,000** | -20,161^*^ | **0,001** |
|  | PB | -7,206 | 0,659 | -4,307 | 1,000 | -3,090 | 1,000 | -17,774^*^ | **0,013** | -14,279^*^ | **0,018** | -29,695^*^ | **0,000** | -25,185^*^ | **0,000** | -30,677^*^ | **0,000** |
|  | | | | | | | | | | | | | | | | | |
| **GL** | SOL | 4,559 | 1,000 | -0,384 | 1,000 | -1,059 | 1,000 | -3,539 | 1,000 | -7,084 | 1,000 | 5,358 | 1,000 | 1,724 | 1,000 | 0,794 | 1,000 |
|  | GM | 2,223 | 1,000 | 3,130 | 1,000 | 1,453 | 1,000 | 2,261 | 1,000 | 5,035 | 1,000 | 12,734 | 0,063 | 11,163^*^ | **0,049** | 11,705 | 0,096 |
|  | TA | -0,719 | 1,000 | -4,639 | 1,000 | -7,748 | 1,000 | 0,469 | 1,000 | -23,823^*^ | **0,000** | -8,803 | 1,000 | -15,189^*^ | **0,018** | -14,316^*^ | **0,006** |
|  | PL | -10,289 | 0,851 | -10,583^*^ | **0,006** | -10,103 | 0,314 | -21,745^*^ | **0,002** | -6,817 | 1,000 | -22,315^*^ | **0,002** | -14,764^*^ | **0,003** | -8,455 | 0,450 |
|  | PB | -4,983 | 1,000 | -1,177 | 1,000 | -1,636 | 1,000 | -15,513^*^ | **0,016** | -9,244 | 0,504 | -16,962^*^ | **0,008** | -14,022^*^ | **0,013** | -18,971^*^ | **0,000** |
|  | | | | | | | | | | | | | | | | | |
| **TA** | SOL | 5,277 | 1,000 | 4,256 | 1,000 | 6,689 | 1,000 | -4,008 | 1,000 | 16,738 | 0,175 | 14,161 | 0,113 | 16,913^*^ | **0,014** | 15,109 | 0,078 |
|  | GM | 2,941 | 1,000 | 7,769 | 0,918 | 9,201 | 0,266 | 1,792 | 1,000 | 28,858^*^ | **0,000** | 21,537^*^ | **0,000** | 26,352^*^ | **0,000** | 26,021^*^ | **0,000** |
|  | GL | 0,719 | 1,000 | 4,639 | 1,000 | 7,748 | 1,000 | -0,469 | 1,000 | 23,823^*^ | **0,000** | 8,803 | 1,000 | 15,189^*^ | **0,018** | 14,316^*^ | **0,006** |
|  | PL | -9,570 | 0,502 | -5,943 | 1,000 | -2,355 | 1,000 | -22,214^*^ | **0,001** | 17,005^*^ | **0,007** | -13,512 | 0,144 | 0,425 | 1,000 | 5,860 | 1,000 |
|  | PB | -4,264 | 1,000 | 3,462 | 1,000 | 6,112 | 1,000 | -15,982^*^ | **0,010** | 14,579^*^ | **0,048** | -8,158 | 1,000 | 1,167 | 1,000 | -4,656 | 1,000 |
|  | | | | | | | | | | | | | | | | | |
| **PL** | SOL | 14,847^*^ | **0,014** | 10,199 | 0,302 | 9,043 | 1,000 | 18,206^*^ | **0,024** | -0,267 | 1,000 | 27,673^*^ | **0,000** | 16,488 | 0,062 | 9,249 | 1,000 |
|  | GM | 12,512 | 0,109 | 13,713^*^ | **0,015** | 11,556 | 0,314 | 24,006^*^ | **0,000** | 11,852 | 0,152 | 35,048^*^ | **0,000** | 25,927^*^ | **0,000** | 20,161^*^ | **0,001** |
|  | GL | 10,289 | 0,851 | 10,583^*^ | **0,006** | 10,103 | 0,314 | 21,745^*^ | **0,002** | 6,817 | 1,000 | 22,315^*^ | **0,002** | 14,764^*^ | **0,003** | 8,455 | 0,450 |
|  | TA | 9,570 | 0,502 | 5,943 | 1,000 | 2,355 | 1,000 | 22,214^*^ | **0,001** | -17,005^*^ | **0,007** | 13,512 | 0,144 | -0,425 | 1,000 | -5,860 | 1,000 |
|  | PB | 5,306 | 1,000 | 9,405^*^ | **0,000** | 8,466 | 0,117 | 6,232 | 1,000 | -2,426 | 1,000 | 5,353 | 1,000 | 0,742 | 1,000 | -10,516^*^ | **0,024** |
|  | | | | | | | | | | | | | | | | | |
| **PB** | SOL | 9,542 | 0,321 | 0,793 | 1,000 | 0,577 | 1,000 | 11,974 | 0,406 | 2,159 | 1,000 | 22,319^*^ | **0,000** | 15,746^*^ | **0,045** | 19,765^*^ | **0,005** |
|  | GM | 7,206 | 0,659 | 4,307 | 1,000 | 3,090 | 1,000 | 17,774^*^ | **0,013** | 14,279^*^ | **0,018** | 29,695^*^ | **0,000** | 25,185^*^ | **0,000** | 30,677^*^ | **0,000** |
|  | GL | 4,983 | 1,000 | 1,177 | 1,000 | 1,636 | 1,000 | 15,513^*^ | **0,016** | 9,244 | 0,504 | 16,962^*^ | **0,008** | 14,022^*^ | **0,013** | 18,971^*^ | **0,000** |
|  | TA | 4,264 | 1,000 | -3,462 | 1,000 | -6,112 | 1,000 | 15,982^*^ | **0,010** | -14,579^*^ | **0,048** | 8,158 | 1,000 | -1,167 | 1,000 | 4,656 | 1,000 |
|  | PL | -5,306 | 1,000 | -9,405^*^ | **0,000** | -8,466 | 0,117 | -6,232 | 1,000 | 2,426 | 1,000 | -5,353 | 1,000 | -0,742 | 1,000 | 10,516^*^ | **0,024** |

Table 6 (supplementary). Significant differences between muscles for each condition with effect sizes (Cohen’s *d*).

| **Configuration** | **Comparisons** | **Difference (%)** | **p-value** | **Effect Size (d)** |
| --- | --- | --- | --- | --- |
| **Floor** | PL vs SOL | 14.85% | p=0.014 | d=0.67 |
| **Rear** | PL vs GM | 13.71% | p=0.015 | d=0.67 |
|  | PL vs GL | 10.58% | p=0.006 | d=0.74 |
|  | PL vs PB | 9.41% | p<0.001 | d=0.90 |
| **Ever** | PL vs SOL | 18.21% | p=0.024 | d=0.64 |
|  | PL vs GM | 24.01% | p<0.001 | d=0.98 |
|  | PL vs GL | 21.45% | p=0.002 | d=0.81 |
|  | PL vs TA | 22.21% | p=0.001 | d=0.89 |
|  | PB vs GM | 17.78% | p=0.013 | d=0.68 |
|  | PB vs GL | 15.51% | p=0.016 | d=0.66 |
|  | PB vs TA | 15.98% | p=0.010 | d=0.69 |
| **Sup** | TA vs GM | 28.86% | p<0.001 | d=1.16 |
|  | TA vs GL | 23.82% | p<0.001 | d=0.95 |
|  | TA vs PL | 17.01% | p=0.007 | d=0.73 |
|  | TA vs PB | 14.58% | p=0.048 | d=0.59 |
|  | PB vs GM | 14.28% | p=0.018 | d=0.66 |
| **Pro** | TA vs GM | 21.54% | p<0.001 | d=1.03 |
|  | PL vs SOL | 27.67% | p<0.001 | d=0.99 |
|  | PL vs GM | 35.05% | p<0.001 | d=1.49 |
|  | PL vs GL | 22.32% | p=0.002 | d=0.80 |
|  | PB vs SOL | 22.32% | p<0.001 | d=0.95 |
|  | PB vs GM | 29.70% | p<0.001 | d=1.21 |
|  | PB vs GL | 16.96% | p=0.008 | d=0.71 |
| **Fore** | GL vs GM | 11.16% | p=0.049 | d=0.59 |
|  | TA vs SOL | 16.91% | p=0.014 | d=0.67 |
|  | TA vs GM | 26.35% | p<0.001 | d=1.35 |
|  | TA vs GL | 14.76% | p=0.018 | d=0.66 |
|  | PL vs GM | 25.93% | p<0.001 | d=1.10 |
|  | PL vs GL | 14.76% | p=0.003 | d=0.77 |
|  | PB vs SOL | 15.75% | p=0.045 | d=0.59 |
|  | PB vs GM | 25.19% | p<0.001 | d=1.01 |
|  | PB vs GL | 14.02% | p=0.013 | d=0.68 |
| **Total** | TA vs GM | 26.02% | p<0.001 | d=1.30 |
|  | TA vs GL | 14.32% | p=0.006 | d=0.74 |
|  | PL vs GM | 20.16% | p=0.001 | d=0.87 |
|  | PB vs SOL | 19.77% | p=0.005 | d=0.75 |
|  | PB vs GM | 30.68% | p<0.001 | d=1.43 |
|  | PB vs GL | 18.97% | p<0.001 | d=1.01 |
|  | PB vs PL | 10.52% | p=0.024 | d=0.64 |
