# Supplementary material for: Comparison of lower-leg muscle activation and establishment of muscle activation patterns during single-leg stance under various instability conditions in healthy active subjects: a cross-sectional study
Source: PeerJ. 2025 May 23;13:e19461. doi: 10.7717/peerj.19461 (PMC12105616; doi:10.7717/peerj.19461)
Supplement: Supplemental Information 4 [file peerj-13-19461-s004.docx]

Variable “sex”:

1. Male
2. Female

Variables “Nor_nEMG_*configuration_muscle*”: normalized value of maximum activation in the indicated *configuration* compared to the maximum activation during the MIVC.

Variables “*Configuration_muscle*_MAX”: maximum activation in the indicated *configuration*.

Variables “*Muscle*_nEMG_MVIC”: maximum activation during the MIVC.
